# Nonstationary Precipitation Intensity-Duration-Frequency Curves for Infrastructure Design in a Changing Climate

**DOI:** 10.1038/srep07093

**Published:** 2014-11-18

**Authors:** Linyin Cheng, Amir AghaKouchak

**Affiliations:** 1 University of California, Irvine, E4130 Engineering Gateway Irvine, CA 92697-2175, USA

## Abstract

Extreme climatic events are growing more severe and frequent, calling into question how prepared our infrastructure is to deal with these changes. Current infrastructure design is primarily based on precipitation Intensity-Duration-Frequency (IDF) curves with the so-called stationary assumption, meaning extremes will not vary significantly over time. However, climate change is expected to alter climatic extremes, a concept termed nonstationarity. Here we show that given nonstationarity, current IDF curves can substantially underestimate precipitation extremes and thus, they may not be suitable for infrastructure design in a changing climate. We show that a stationary climate assumption may lead to underestimation of extreme precipitation by as much as 60%, which increases the flood risk and failure risk in infrastructure systems. We then present a generalized framework for estimating nonstationary IDF curves and their uncertainties using Bayesian inference. The methodology can potentially be integrated in future design concepts.

Human activities in the past century have caused an increase in global temperature^1^,^2^,^3^. The rising temperatures boost the atmosphere's water holding capacity by about 7% per 1°C warming, thus directly affecting precipitation^4^. Higher atmospheric water vapor can lead to more intense precipitation events^5^,^6^. Furthermore, rising temperatures and subsequent increases in atmospheric moisture content may increase the probable maximum precipitation (PMP) or the expected extreme precipitation^5^. Consequentially, global warming increases the risk of climatic extremes^1^,^7^,^8^ including floods and damage to infrastructure such as dams, roads and sewer and storm water drainage systems^9^,^10^. Indeed, ground-based observations in the U.S. show a substantial increase in extreme rainfall events during the past century^1^,^7^,^11^,^12^,^13^,^14^,^15^. Global-scale studies also show increased precipitation in northern Australia, central Africa, Central America and parts of southwest Asia^16^.

Current infrastructure design concepts to deal with flooding and precipitation are based on local rainfall Intensity-Duration-Frequency (IDF) curves. These curves are widely used in municipal storm water management and other engineering design applications across the world^17^,^18^,^19^,^20^,^21^,^22^,^23^,^24^,^25^. The IDF curves are based on historical rainfall time series data and designed to capture the intensity and frequency of precipitation for different durations. Rainfall intensities corresponding to particular durations (e.g., 1-hr, 2-hr, 6-hr, 24-hr) are obtained by fitting a theoretical probability distribution to annual extreme rainfall. Current IDF curves are based on the concept of temporal stationarity, which assumes that the occurrence probability of extreme precipitation events is not expected to change significantly over time^17^,^26^. However, climate change is expected to alter the intensity, duration or frequency of climatic extremes over time. This change is termed nonstationarity in the literature^26^,^27^,^28^,^29^.

Given the observed increase in heavy precipitation events, we argue that the IDF curves should be updated to account for a changing climate, especially for urban infrastructure design^17^. There are many studies on changes in precipitation intensity or frequency and also multivariate frequency analysis^18^,^30^,^31^,^32^,^33^,^34^,^35^,^36^,^37^,^38^. However, methods for assessing changes in precipitation intensity, duration and frequency and their uncertainty in a nonstationary climate are limited. Climate-related nonstationarity in a time series of precipitation extremes can be associated with climate variability as well as climate change^39^. There are different parametric and non-parametric methods to address nonstationarity in time series^40^,^41^,^42^,^43^,^44^,^45^,^46^,^47^,^48^. In this paper, we assess the effect of nonstationarity on IDF curves and the occurrence of extremes. We also outline a generalized framework for constructing IDF curves under nonstationary conditions. The fundamental concept is based on the Generalized Extreme Value distribution (GEV) combined with Bayesian inference for uncertainty assessment (see Methods Section).

Our analyses are based on ground-based observations of precipitation extremes (here, annual maxima) from the United States National Oceanic and Atmospheric Administration Atlas 14, the basis for IDF curves in the United States^49^. Following the NOAA Atlas 14 approach, the annual maxima series is constructed by extracting the highest precipitation amount for different precipitation durations (e.g., 1-hr, 6-hr) in each successive water year (i.e., 12-month period beginning October 1 and continuing through September 30). While previous studies show that precipitation extremes in several regions have increased, still most ground-based stations do not exhibit a strong trend and only limited stations show a statistically significant nonstationary behavior^50^. To examine the effect of ignoring nonstationarity, historical rainfall data (1949–2000), used in the updated NOAA Atlas 14, from five stations that exhibit significant increasing trends are used to assess IDF curves under nonstationarity (Table 1). In the selected stations and based on the Mann-Kendall trend test, precipitation extremes exhibit nonstationary behavior for different durations (here, 1-hr to 96-hr) at 95% confidence level (Figure S1 in Supplementary Material). The presence of a statistically significant increasing trend in precipitation extremes violates the basic assumption of stationary IDF curves.

A unique feature of this modeling framework is that it offers uncertainty bounds of IDF estimates based on a Bayesian approach (see Equations 3 and 4 in Methods Section). To illustrate, the stationary and nonstationary IDF curves for different return periods (2-, 10-, 25-, 50- and 100-year) and durations for the White Sands National Monument Station in New Mexico are presented in Figure 1. As discussed in Method Section, along with model parameters, the uncertainty bounds of IDF curves can be obtained simultaneously in the proposed framework. The gray lines show the uncertainty bounds of the nonstationary IDF estimates based on the Differential Evolutionary Monte Carlo algorithm built into a Bayesian framework^51^ for extreme value analysis (Methods Section). This Bayesian framework has several advantages over the commonly used frequentist inference^52^. For example, a Bayesian framework allows using priors (or results from a previous/similar model) to inform model parameter inference (e.g., priors derived from a nearby or similar station with longer records to improve inferences at a location with a shorter record). Furthermore, Bayesian inference offers uncertainty of the parameter estimates, yielding more realistic estimations^53^. For more information on both Bayesian and frequentist options the interested reader is referred to Bernardo and Smith^52^ and Press^54^.

We found that the stationary assumption delivers IDF curves that can substantially underestimate extreme events. If such a stationary IDF curve is used for an infrastructure design, the project may not be able to withstand more extreme events, which are shown by nonstationary estimates for the same return period. For example, for a 2-year 2-hr storm (i.e., an event with a return period of 2 years and duration of 2 hours), the difference between the nonstationarity (14.7 mm/hr) and stationarity (9.1 mm/hr) extreme precipitation is about 5.6 mm/hr (+61.5%); while for a 10-year 1-hr event, the difference between nonstationarity (35.0 mm/hr) and stationarity (25.9 mm/hr) extreme precipitation is over 9.1 mm/hr (+35.1%). Even for a small watershed, this extra 9.1 mm/hr (+35.1%) precipitation would lead to a significant increase in flood peak. In other words, a stationary assumption will underestimate the peak flood and as a result the actual flood risk will be higher than what the system or infrastructure is designed for.

The differences between the nonstationary and stationary estimates decrease for longer durations (e.g., 168-hr precipitation). This implies that in this station, shorter precipitation events have been intensified more in the past decades, while longer events have not changed substantially. For the same station shown in Figure 1, the boxplots of differences between the nonstationary and stationary precipitation extremes are presented in Figure 2. The figure shows that for all durations and return periods, the quantile boxes are above zero, indicating underestimation of extremes in a stationary assumption. In all durations, the uncertainty increases as the return period increases. Consequently, the uncertainties in the stationary and nonstationary precipitation differences increase at higher return periods.

By examining storm durations, we found that the shorter the duration the larger the differences between the nonstationary and stationary extremes. As an example, for the 100-year return period, the differences between nonstationary and stationary IDF curves of 1-hr and 2-hr events reduce from 4.7–15.6 mm/hr to 1.4–7.3 mm/hr, while for a 168-hr storm, the difference approaches zero (see Figure 2). Similar behavior is observed in the other stations and as a result we have focused on shorter durations. For the other stations in Nevada (NV), California (CA) and North Carolina (NC), Figure 3 summarizes the differences between the stationary and nonstationary precipitation extremes for different return periods and durations (top two rows: 1-hr duration; bottom two rows: 2-hr duration). The boxplots show the median (center mark), and the 25th (lower edge) and 75th (upper edge) percentiles of the differences between stationary and nonstationary estimates. Similar behavior emerges in different stations, indicating that nonstationary estimates are larger than their corresponding stationary values. Such difference in underestimation raises the risk of extreme floods and damage to infrastructure if nonstationarity is ignored^55^.

Infrastructure health and safety during precipitation extremes is closely related to human health and security, particularly downstream of major structures (e.g., dams, spillways, reservoirs). For this reason, methods that can account for changing precipitation extremes are essential for updating engineering standards and design codes. Potential non-uniform and climate-induced changes on heavy rainfall events calls into question the accuracy and adequacy of current infrastructure design concepts, which rely on an assumption of climate stationarity. Previous studies show that limited locations across the United States exhibit a strong nonstationary increase in precipitation extremes. The reported underestimation of extremes corresponds to the few stations investigated in this paper that exhibit a strong nonstationary increase in precipitation extremes. While still limited locations exhibit a statistically significant increasing trend in precipitation extremes, future projections indicate that extremes may become more frequent. We show that ignoring the nonstationary assumption could lead to substantial underestimation of extremes, especially at sub-daily durations (e.g., 1-hr, 2-hr).

We outline a novel framework to create the next generation of IDF curves to be incorporated into infrastructure design. This paper does not argue that the proposed methodology should be used for all stations. In fact, the suggested nonstationary IDF curve estimation method would only be necessary when observations or model projections indicate a nonstationary future climate. If there is no indication of change in historical records or model simulations, current stationary IDF curve models may be sufficient for infrastructure design. The presented methodology can be used to improve our understanding of the climate-induced changes in the intensity, duration and frequency of heavy precipitation, and can potentially be integrated in design concepts. However, infrastructure design and construction require substantial investment over a long period of time and effective integration of this methodology as well as development of adaptive design frameworks will require collaborative and interdisciplinary research with engineers, policy makers, economists, climate scientists and decision makers.

Finally, given that IDF curves have been widely used for design and practical applications, this study focuses on a nonstationary framework that still relies on the IDF concept. Alternative methods such as the stochastic storm transposition method offer a different way forward for flood risk assessment^56^,^57^. However, incorporating nonstationarity in these methods deserves a great deal of research. We predict that in future more research will be dedicated to develop more physically-based nonstationary frameworks for flood risk assessment.

## Methods

The GEV distribution is a combination of Gumbel, Fréchet, and Weibull distributions, and is based on the limit theorems for block maxima or annual maxima^58^. The standard cumulative distribution of the GEV can be expressed as^59^: 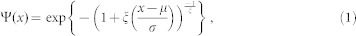
where ψ(*x*) is defined for 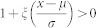; elsewhere, ψ(*x*) is either 0 or 1^60^. The GEV distribution has the location parameter (*μ*), the scale parameter (*σ*) and the shape parameter (*ξ*) to specify the center of the distribution, the deviation around *μ*, and the tail behavior of the GEV distribution, respectively. For *ξ*→0, *ξ* < 0, and *ξ* > 0, the GEV leads to the Gumbel, Weilbull and Fréchet distributions, respectively.

The extreme value theory of stationary random sequences assumes that statistical properties of extremes (here, distribution parameters *θ* = (*μ*, *σ*, *ξ*) are independent of time^51^. In a nonstationary process, however, the parameters of the underlying distribution function are time-dependent and the properties of the distribution would therefore vary with time^61^. As in previous studies, the location parameter (*μ*) is assumed to be a function of time (Equation 2), while keeping the scale and shape parameters constant^51^,^58^,^62^,^63^: 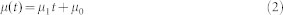
where *t* is time, and *β* = (*μ*_1_, *μ*_0_) are the regression parameters.

The non-parametric rank based Mann-Kendal (MK) test is used to detect trends in annual maximum rainfall intensity of the selected storm durations (i.e., 1, 2, 3, 6, 12, 24, 48 and 168 hours). The null hypothesis of no trend is rejected if the test statistic is significantly different from zero at a 0.05 significance level. Upon detection of a significant trend, the location parameter will be computed under the nonstationary assumption (Equation 2). This will allow estimating rainfall quantities in a more realistic way consistent with the behavior of observed precipitation extremes. It is worth pointing out that the purpose including the MK test is solely to avoid implementing a time varying extreme value analysis on a data that does not show a significant change in extremes over time. Technically, the same methodology can be applied to all data sets regardless of their trend, avoiding a subjective significance measure. In fact, recent studies highlight the need to go beyond subjective criteria for significance analysis, especially in a nonstationary world^64^.

Providing uncertainty estimates for IDF curves is fundamental to risk assessment and decision making. In this study, a Bayesian-based Markov chain approach is integrated into the nonstationary GEV for uncertainty assessment^65^. This approach combines the knowledge brought by a prior distribution and the observation vector 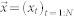 of *N* annual maxima (Equations 3 and 4) into the posterior distribution of parameters *θ* = (*μ*, *σ*, *ξ*). Assuming independence between observations, the Bayes theorem for estimation of GEV parameters under the nonstationary assumption can be expressed as^51^,^63^: 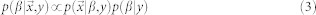

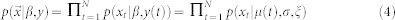
where *y*(*t*) denotes the set of all covariate values under the nonstationary assumption, and stationarity can be treated as a special case without the term *y*(*t*). The resulting posterior distributions 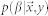 provide information on the distribution parameters (*μ*_1_, *μ*_0_, *σ*, *ξ*).

To estimate the parameters inferred by Bayes, the Differential Evolution Markov Chain (DE-MC) is integrated to generate a large number of realizations from the parameters' posterior distributions^66^,^67^. The DE-MC attributes to the genetic algorithm Differential Evolution (DE)^66^,^67^ for global optimization over real parameter space with the Markov Chain Monte Carlo (MCMC) approach. In this model, the target posterior distributions are sampled through five Markov Chains constructed in parallel. These chains are allowed to learn from each other by generating candidate draws based on two random parent Markov Chains (rather than to run independently^66^); therefore, it has advantages of simplicity, speed of calculation, and convergence over the conventional MCMC^66^. By combining DE-MC with Bayesian inference, the confidence interval and uncertainty bounds of estimated return levels based on the sampled parameters can be obtained simultaneously for IDF curves. The probabilistic nature of the methodology allows investigating the posterior histograms of the model parameters and sample regression parameters. For example, the sampled regression parameters of *μ*_0_ and *μ*_1_ for White Sands National Monument Station in New Mexico are presented in Supplementary Material Figures S2 and S3, respectively.

For a time series of annual maxima, the time-variant parameter (*μ*(*t*)) is derived by computing the 95th percentile of DE-MC sampled *μ*(*t*), termed as 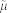 (i.e., 95th percentile of *μ*(*t = 1*),…, *μ*(*t = 100*)). The model parameters will then be used to estimate the nonstationary precipitation intensity (or equivalently, return level) analogous to the standard ones as follows: 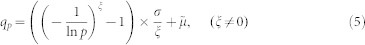
Where *p* (the non-exceedance probability of occurrence) is frequency, i.e., how often a storm of specified intensity and duration is expected to occur. The *n*-year precipitation intensity refers to the annual maximum rainfall of specified depth/intensity and duration having a probability of exceedance of *1/n*. In this approach, precipitation intensity is expressed as a function of the return period *T*, 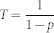, i.e., the average length of time between events of a given depth/intensity and duration.

## Author Contributions

L.C. and A.A. conceived the study. L.C. analyzed the data and performed the experiment. A.A. and L.C. wrote the paper. All authors reviewed the manuscript.

## Supplementary Material

Supplementary Information Supplementary Material

## Figures and Tables

**Figure 1:**
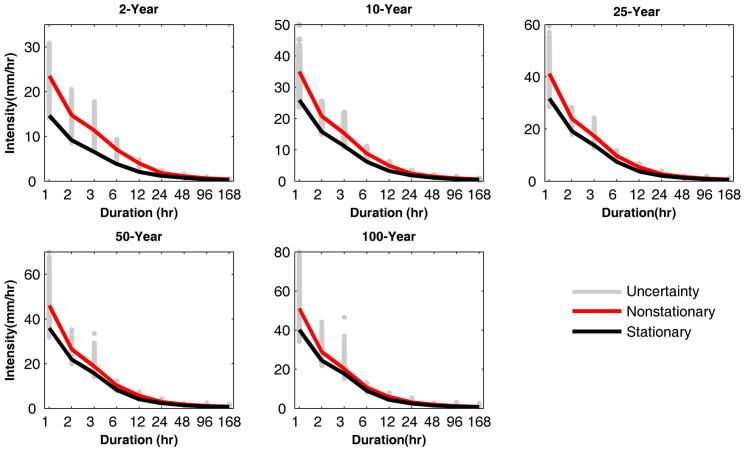
Nonstationary vs. stationary IDF Curves for different return periods and durations at the selected station in White Sands National Monument Station, New Mexico. The stationary assumption consistently underestimates the IDF curves over different durations. The gray area shows the uncertainty bound of nonstationary IDF estimates (figure generated using Matlab®).

**Figure 2:**
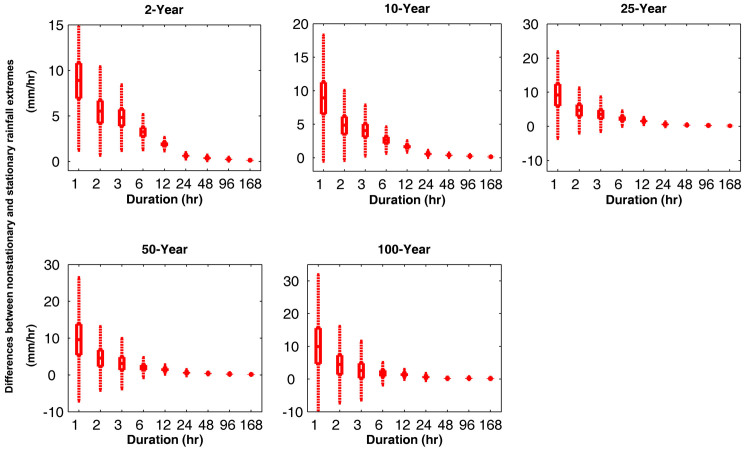
Differences between the nonstationary and stationary precipitation extremes for different return periods and durations in White Sands National Monument Station, New Mexico. The boxplots show the median (center mark), and the 25^th^ (lower edge) and 75^th^ (upper edge) percentiles (figure generated using Matlab®).

**Figure 3:**
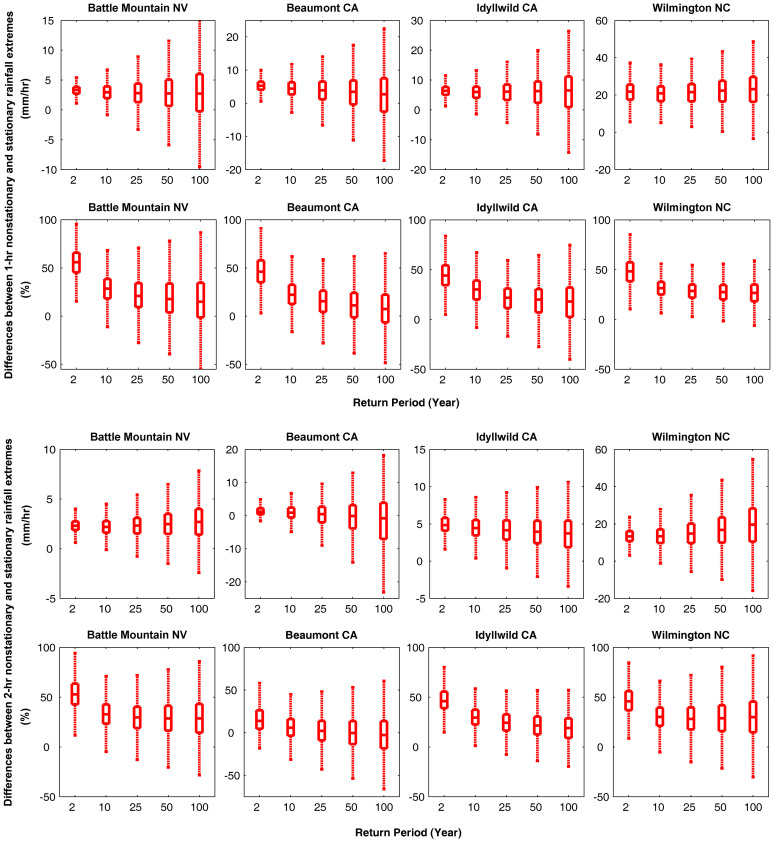
Difference between the nonstationary and stationary precipitation extremes estimates for different return periods and durations (top two rows: 1-hr duration; bottom two rows: 2-hr duration) in mm/hr and percent (%). The boxplots show the median (center mark), and the 25^th^ (lower edge) and 75^th^ (upper edge) percentiles (figure generated using Matlab®).

**Table 1:** Selected stations for analysis of Intensity-Duration-Frequency curve analysis under stationary and nonstationary assumptions, and their average annual maxima (AAM) for selected precipitation durations

				AAM (mm)		
Station Name	State	Latitude	Longitude	1-hr	2-hr	6-hr
White Sands National **M**onument	**NM**	**32.7817**	**106.1747**	**16.83**	**19.87**	**24.22**
Battle Mountain	**NV**	**40.6167**	**116.8667**	**6.65**	**9.16**	**14.60**
Beaumont	**CA**	**33.9292**	**116.9750**	**13.28**	**19.63**	**34.19**
Idyllwild Fire Dept	**CA**	**33.7472**	**116.7144**	**15.83**	**21.70**	**39.97**
Wilmington WSO Airport	**NC**	**34.2683**	**77.9061**	**46.26**	**61.22**	**86.00**
