# Supplementary material for: Nonstationary Precipitation Intensity-Duration-Frequency Curves for Infrastructure Design in a Changing Climate
Source: Sci Rep. 2014 Nov 18;4:7093. doi: 10.1038/srep07093 (PMC4235283; doi:10.1038/srep07093)
Supplement: Supplementary Information — Supplementary Material [file srep07093-s1.pdf]

Supplementary Material

Nonstationary Precipitation Intensity-Duration-Frequency Curves for Infrastructure Design in a Changing Climate

Linyin Cheng, and Amir AghaKouchak*

University of California, Irvine, E4130 Engineering Gateway Irvine, CA 92697-2175, USA

* Corresponding Author: Amir AghaKouchak (amir.a@uci.edu)

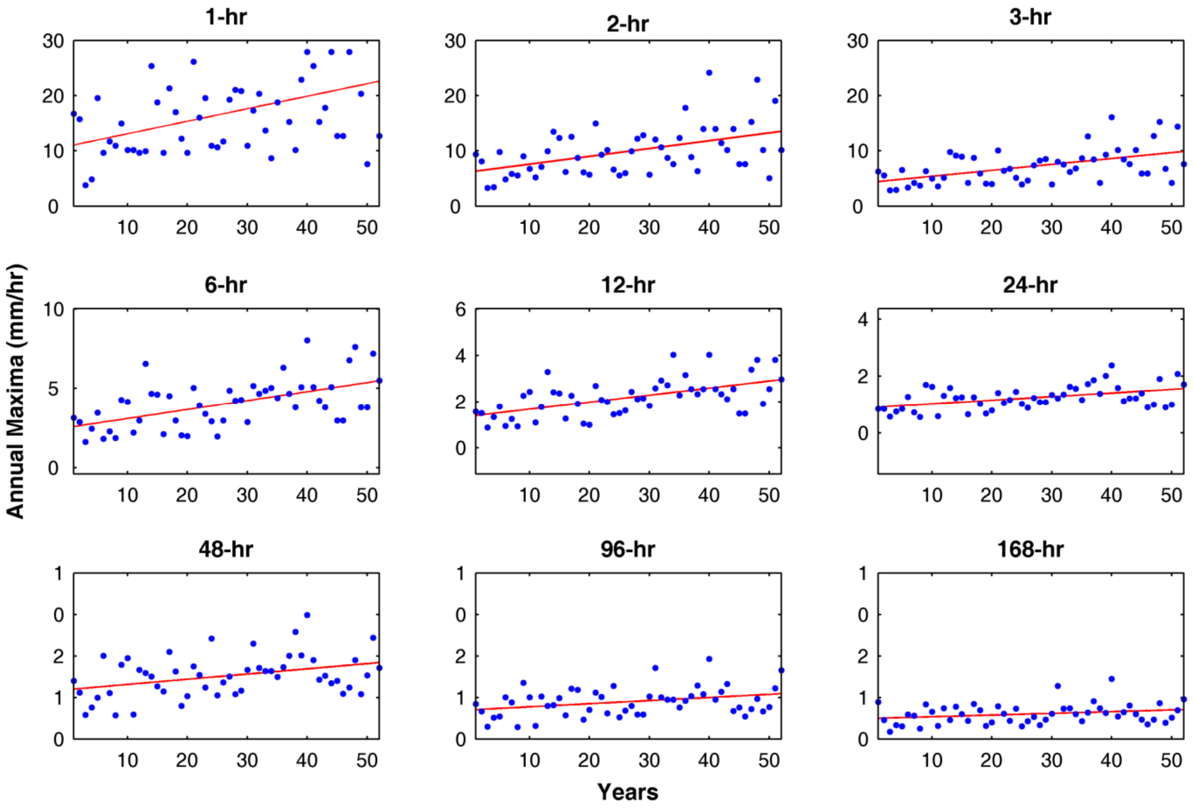

Figure S1 - Significant trends in annual maxima precipitation over different durations in the selected station in White Sands National Monument, New Mexico (latitude 40.62^{o}, longitude 116.87^{o}) - figure generated using Matlab®.

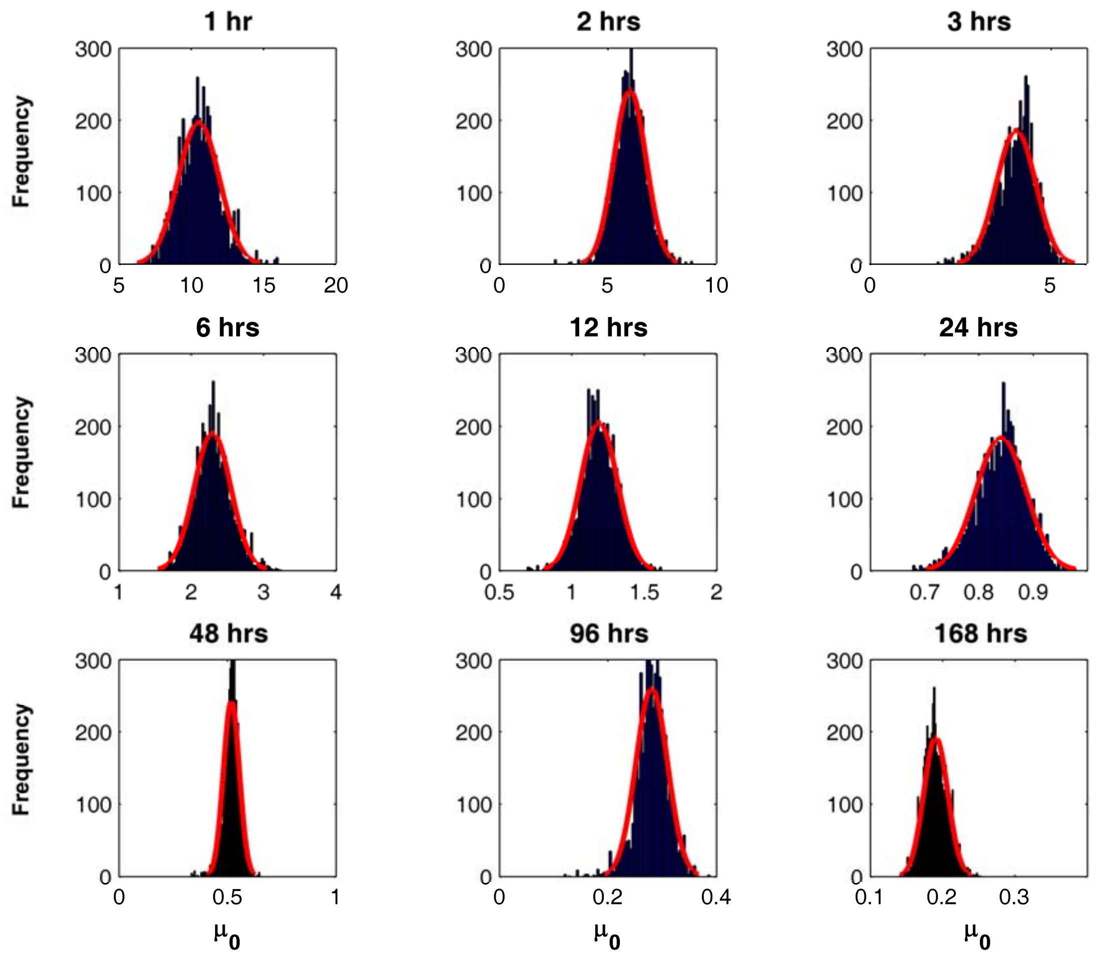

Figure S2 - Posterior distribution of the regression parameter μ_0 in the selected station in White Sands National Monument Station, New Mexico (latitude 40.62°, longitude 116.87°) - figure generated using Matlab®.

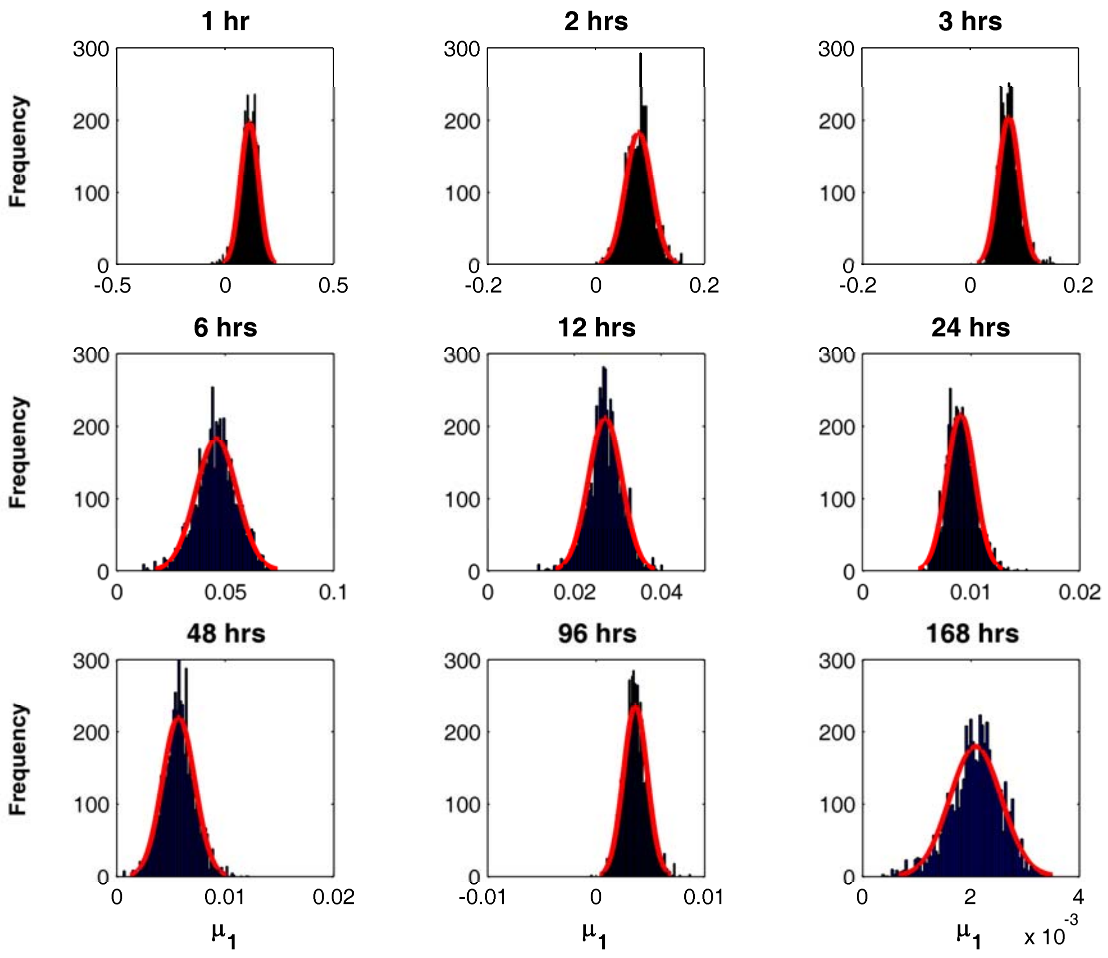

Figure S3 - Posterior distribution of the regression parameter μ_1 in the selected station in White Sands National Monument Station, New Mexico (latitude 40.62°, longitude 116.87°) - figure generated using Matlab®.
